# Surgeons’ preferences for using sentinel lymph node biopsy in patients with ductal carcinoma in situ

**DOI:** 10.1371/journal.pone.0269551

**Published:** 2022-06-06

**Authors:** Claudia J. C. Meurs, Janine A. van Til, Marian B. E. Menke-Pluijmers, Stefanie de Vet, Sabine Siesling, Pieter J. Westenend

**Affiliations:** 1 CMAnalyzing, Zevenaar, the Netherlands; 2 Department of Health Technology and Services Research, Technical Medical Centre, University of Twente, Enschede, the Netherlands; 3 Department of Surgery, Albert Schweitzer Hospital, Dordrecht, the Netherlands; 4 Department of Research and Development, Netherlands Comprehensive Cancer Centre Organisation, Utrecht, the Netherlands; 5 Laboratory of Pathology Dordrecht, Dordrecht, the Netherlands; University of Limerick, IRELAND

## Abstract

**Background:**

There is a large variation between Dutch hospitals in the use of Sentinel Lymph Node Biopsy (SLNB) in patients with a biopsy diagnosis of Ductal Carcinoma in Situ. The aim of our study was to investigate whether this variation might be explained by preferences of surgeons, organisational factors or the influence of patients preferences.

**Methods:**

A cross-sectional web survey was conducted among 260 Dutch oncological/breast surgeons. Preferences of surgeons and the influence of the patients’ preferences were determined by means of best-worst scaling (BWS) of profile case scenarios and by ranking risk factors. The survey also explored organisational questions, the reported use of diagnostic techniques and influences on the decision.

**Results:**

The BWS scenarios were completed by 57 surgeons. The most important reasons for performing SLNB were a suspected invasive component and DCIS grade 3. In the ranking, these were also the first and second most important factor, followed by the size of the lesion and a mass on mammogram. In 58% to 70% of the scenarios, the surgeons would not change their decisions on the use of SLNB if the patient’s chose differed. No organisational factor was significantly associated with the reported use of SLNB.

**Conclusion:**

The inter-hospital variation in the use of SLNB could not be attributed to organisational factors or surgeons’ preferences for risk factors. The risk factors that most surgeons reported as reasons for performing SLNB are consistent with the factors described in the Dutch treatment guideline for the use of SLNB.

## Introduction

Of the patients with a preoperative diagnosis of Ductal Carcinoma in Situ (DCIS), 20 to 25% will have a final diagnosis of invasive breast cancer after surgery [1–3]. For this reason, staging with Sentinel Lymph Node Biopsy (SLNB) is considered in patients thought to be at high risk of a diagnosis of invasive breast cancer. Several international guidelines have been developed to select patients with an increased risk [4, 5]. Previous studies, showed differences between hospitals in the use of axillary evaluation or SLNB for DCIS [6, 7].

It can be hypothesized that variations between hospitals are associated with differences in surgeons interpretation of the treatment guidelines and in their level of agreement with these guidelines. Survey studies from the UK [7, 8] have shown that the opinions of surgeons deviated from the NICE guidelines, which state that extensive microcalcification or a palpable mass are indications for SLNB in case of breast conserving surgery [5].

For the Netherlands, no data is available on diverging opinions of surgeons. Also, the variation might be influenced by differences in how hospitals have organised the care for patients with DCIS, for instance how often they use a vacuum-assisted device as a diagnostic technique. Furthermore, it is unknown to what extent the patient has an influence on the decision to use SLNB. We did not find studies that investigated these other potential reasons for variations between hospitals in the use of SLNB.

The aim of our study was to determine whether the inter-hospital variation in the use of SLNB in the Netherlands could be explained by preferences of surgeons, organisational factors or the influence of patient.

## Methods

### Survey

A cross sectional web survey was done in the Netherlands. A total of 260 surgeons, from 77 hospitals were invited by e-mail. Two reminders were send. The survey instrument consisted of best-worst scaling (BWS) scenarios, questions on ranking of patient and tumour characteristics, background information and organisational factors (see S1 File). The BWS scenarios and the ranking questions were preference questions and therefore answers were included of all surgeons who completed the survey. For the background characteristics and the organizational factors we used the answers of the first surgeon of a specific hospital who completed the survey. We aimed at a response rate of 30% by individual respondents covering at least 50% of the invited hospitals. The survey was done online with the program LimeSurvey.

### Best-worst scaling scenarios

A BWS case 2 format was used to determine which risk factors were important in the decision to use SLNB. The following attributes and levels were selected based on the literature on risk factors for invasive breast cancer after a DCIS diagnosis and on treatment guidelines:

- Age: < 55 / 55 to 70 / > 70 years
- DCIS grade: grade 1 / grade 2 / grade 3
- Size of the lesion on the mammogram: ≤ 2 cm / > 2cm
- Suspected invasive component on biopsy: yes / no
- BI-RAD score: score 4 / score 5
- Palpability of the lesion: yes / no

Based on the attribute level combinations, 144 profiles were constructed with the software Sawtooth 6.4.6. The attributes vary from two to three levels, therefore the number of times a level is included in the survey is not equal and the combination of levels is also not equal, meaning that the BWS design is not balanced and not orthogonal. Of all possible profiles: 32 were selected and used in a block design; four survey designs and each respondent had to answer questions regarding 8 scenarios. The case 2 BWS design is a profile case design and thus only one scenario is shown at a time. Each scenario had to be completed twice: once for breast conserving surgery and once for mastectomy. When starting the online survey, the respondent was allocated to one of the four survey designs. For each scenario, the respondent was asked to select the least important and the most important risk factor (attribute/level combination), assuming that SLNB will be done. Secondly, for each scenario the respondent was asked whether they would perform SLNB in that scenario, and thirdly, whether they would change their decision in case the patient whished the opposite option. The BWS scenarios of one of the survey designs is given in S1 File.

### Ranking

Respondents were also asked to rank the following 12 factors on the degree of importance and on their influence on the decision to perform SLNB:

- All the attributes of the BWS (see above)
- Type of surgery: breast conserving surgery or mastectomy
- Contralateral tumour: presence or absence
- Direct reconstruction of the breast during surgery: yes or no
- Multifocal or multicentric
- Breast cancer screening: found within or outside
- Solid component on mammogram: yes or no

### Organisational factors

Surgeons answered questions about background characteristics, such as the availability of nuclear medicine. Also, questions were asked about organisational factors such as, the use of diagnostic techniques, how often SLNB was used and how the decision to use SLNB in individual patients was made. The answer options the questions about organisational factors were provided on a 5-point Likert scale: never, sometimes, regularly, often and always.

### Pilot

The survey was pre-tested with 10 persons. Based on their feedback, the formulation was changed for questions that were unclear or ambiguous. Also, the test panel found the survey quite time-consuming, hence the attributes in the scenarios were placed in a fixed order to make the scenarios easier to read. Furthermore, the test panel considered not all patient scenarios clinically realistic. Therefore, the selected levels in some of the selected scenarios were altered to construct patient scenarios that were clinically realistic. The patient scenarios that were used in the study are described in S2 File.

### Analysis

Associations between the reported use of SLNB and background characteristics and organisational factors were tested with Fisher’s exact test. A Bonferroni correction was applied on the level of significance; since 26 tests were done, a p-value of less than 0.002 was considered significant. This analysis was done with STATA statistics. For the BWS analysis, it was counted how often a particular attribute-level combination was selected as the most important factor and how often it was selected as the least important factor; next, the difference of best-minus-worst was calculated. This BW score was standardized to correct for the unbalanced design by dividing the score by the maximum number of times an attribute-level combination was presented to the respondents. The possible BW scores ranged from -1.0 to 1.0, with a positive score meaning that the risk factor was selected more often as the most important factor than it was selected as the least important factor and a negative score meaning the opposite. For the ranking of the risk factors, the scores were calculated with the rank sum weights method: n-r+1, where n is the total number of factors and r is the rank a respondent has given to a factor. For example, the factor that was selected as the second most important factor was scored 11: 12–2+1. The factors were ranked on the total score they received.

## Results

### Respondents characteristics

The questions about the background characteristics and the organisational factors were filled out by 81 surgeons (of 260, 31%) representing 57 different hospitals (of 77, response rate 74%). Of these, 24 were excluded since only one questionnaire on organisational factors per hospital was included. Table 1 shows the background characteristics that were provided by surgeons, representing 57 hospitals.

**Table 1:** Respondent characteristics of the first responding surgeon of each hospital.

Respondent characteristics	N	%
Gender		
Male	30	53%
Female	27	47%
Specialty		
General surgeon	0	0%
Oncological surgeon	57	100%
Number of years working in your specialty		
0 to 5 years	5	9%
5 to 10 years	24	42%
10 to 15 years	9	16%
More than 15 years	19	33%
Number of patients with DCIS each year in your hospital		
Fewer than 20 patients per year	9	16%
20 to 30 patients per year	16	28%
More than 30 patients per year	32	56%
Technique used to identify sentinel lymph node		
Patent blue	0	0%
Radioactive technetium	10	18%
Radioactive technetium and patent blue	47	82%
Availability of nuclear medicine		
Yes	44	77%
No	0	0%
No, but we have a partnership with a hospital which offers this	13	23%

### Best-worst scaling scenarios

In total, 57 of 260 surgeons answered the BWS questions, resulting in a response rate of 22%. ‘Having a suspected invasive component’ and ‘grade 3 DCIS’ were selected most frequently as the most important factor for performing SLNB. Suspected invasive component and grade also had the highest differences in the ratios between the levels, which indicates that these attributes have the greatest impact on the decision to perform SLNB (see Table 2a and 2b). Size of the lesion on mammogram of > 2 cm also had a positive BW ratio. This ratio was lower because the size was selected less often as the most important factor and because it was also sometimes selected as the least important factor in a surgeon’s motivation for performing SLNB. All levels of the attributes age, palpability and BI-RADS score were selected more often as the least important risk factor than as the most important risk factor, resulting in a negative BW ratio.

**Table 2:** 

**a: Best-worst scaling for breast conserving surgery**						
	Maximum^#^	Most important		Least important		Best-Minus-worst
	N	n	ratio	n	ratio	difference in ratio
Having a suspected invasive component on biopsy	228	173	0,76	2	0,01	0,75
DCIS grade 3	155	65	0,42	0	0,00	0,42
DCIS grade 1	143	49	0,34	14	0,10	0,24
Size of the lesion on mammogram >2 cm	227	36	0,16	6	0,03	0,13
DCIS grade 2	158	23	0,15	9	0,06	0,09
No suspected invasive component on biopsy	228	32	0,14	16	0,07	0,07
Size of the lesion on mammogram < = 2 cm	229	14	0,06	21	0,09	-0,03
Lesion is palpable	176	32	0,18	43	0,24	-0,06
Age of the patient < 55 years	158	5	0,03	22	0,14	-0,11
BI-RADS score 5	228	12	0,05	41	0,18	-0,13
BI-RADS score 4	228	3	0,01	61	0,27	-0,25
Lesion is not palpable	215	2	0,01	69	0,32	-0,31
Age of the patient between 55 and 70 years	152	1	0,01	60	0,39	-0,39
Age of the patient > 70 years	146	2	0,01	67	0,46	-0,45
**b: Best worst scaling for mastectomy**						
	Maximum^#^	Most important		Least important		Best-Minus-Worst
	n	n	ratio	n	ratio	difference in ratio
Having a suspected invasive component on biopsy	241	193	0,80	0	0,00	0,80
DCIS grade 3	155	81	0,52	0	0,00	0,52
DCIS grade 1	143	54	0,38	11	0,08	0,30
DCIS grade 2	158	27	0,17	4	0,03	0,15
Size of the lesion on mammogram >2 cm	227	29	0,13	6	0,03	0,10
No suspected invasive component on biopsy	215	7	0,03	5	0,02	0,01
BI-RADS score 5	228	6	0,03	24	0,11	-0,08
Age of the patient < 55 years	158	6	0,04	24	0,15	-0,11
Size of the lesion on mammogram < = 2 cm	159	10	0,06	27	0,17	-0,11
Lesion is palpable	176	30	0,17	53	0,30	-0,13
BI-RADS score 4	228	1	0,00	55	0,24	-0,24
Lesion is not palpable	215	0	0,00	76	0,35	-0,35
Age of the patient more than 70 years	146	2	0,01	56	0,38	-0,37
Age of the patient between 55 and 70 years	153	3	0,02	68	0,44	-0,42

^#^ The number of times the level was part of a scenario

The question “Would you perform the SLNB” was answered with “yes” for 395 of 456 of the mastectomy scenarios (87%) for 340 of 456 of the BCS scenarios (75%). The answers for each scenario is given in S3 File.

### Ranking

In total, 55 respondents (21%) completed the ranking of the risk factors. ‘Suspected invasive component on biopsy’ and ‘DCIS grade on biopsy’ were the factors with the highest score (see Table 3). The respondents had the option to mention other risk factors that we missed in the ranking. MRI was mentioned twice and the wish of the patient three times.

**Table 3:** Ranking of topics.

Topic	Score
Suspected invasive component on biopsy: yes or no	627
DCIS grade	592
Size of the lesion on mammogram	444
Solid component on mammogram: yes or no	417
Type of operation: breast conserving surgery or mastectomy	363
Palpable: yes/no	360
Multifocal or multicentric	354
Age	287
BI-RADS score	267
Contralateral tumour: present or absent	205
Direct reconstruction of the breast during surgery: yes or no	201
Found by means of breast cancer screening or not	173

### Influence of the patient

**Fig 1:**
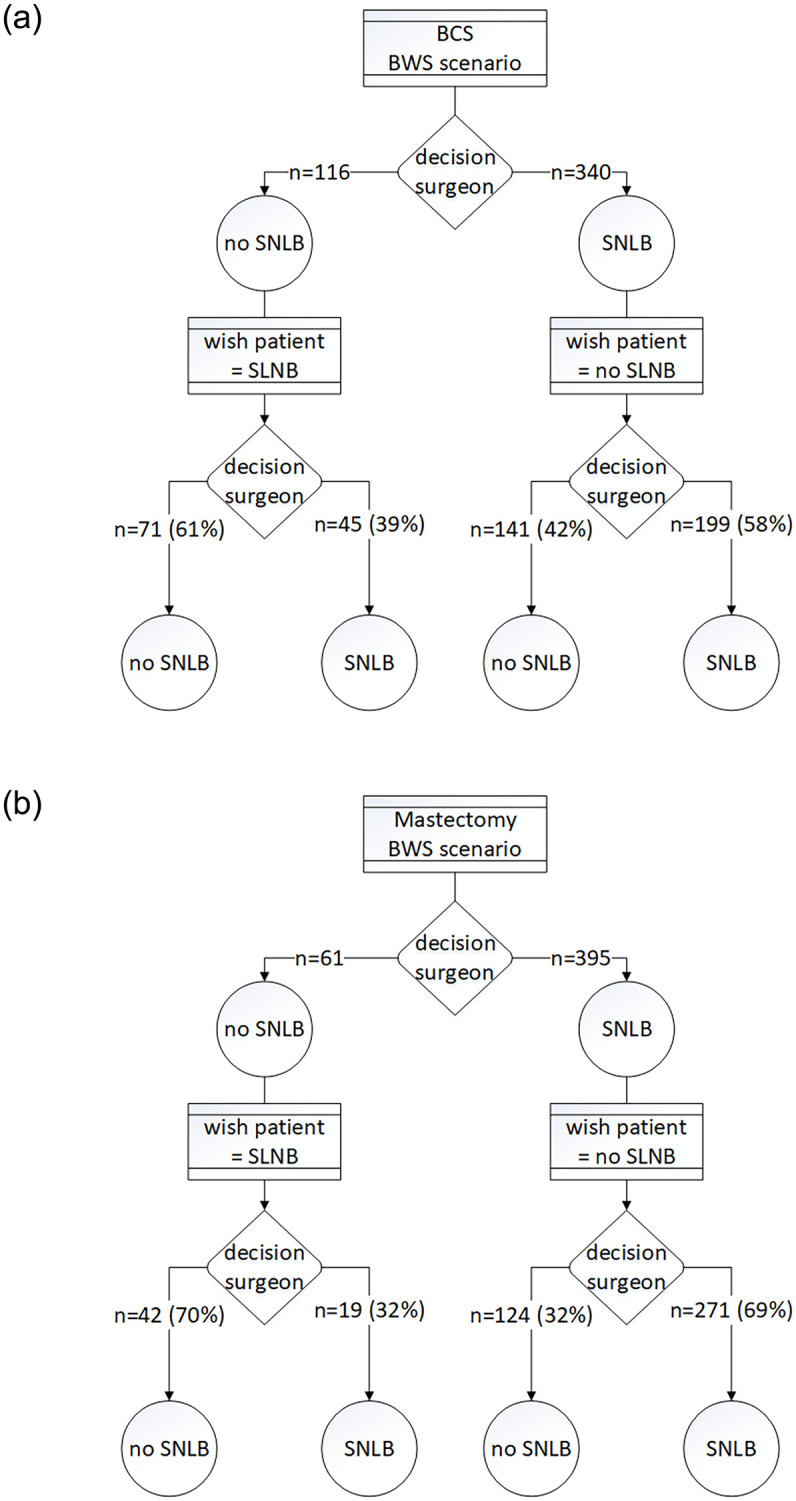
Influence of the patient’s wish on the decision to perform SLNB; 1a: for breast conserving surgery. 1b: for mastectomy.

For a total of 912 patient scenarios, the respondents were asked whether they would change their decision if they were informed that the patient would like the opposite (see Fig 1a and 1b and S3 File). Of the surgeons who chose SNLB in a breast conserving surgery scenario, 58% replied that they would still perform SLNB even if the patient wished otherwise, and so did 69% of the surgeons who chose SNLB in a mastectomy scenario.

### Organisational factors

The answers of surgeons representing 57 of the 77 hospitals were used in the analyses of the organisational factors, the response rate was 74% (see Table 4).

**Table 4:** Assessment of the organisational factors.

	Always		Often		Regular		Sometimes		Never		Not applicable	
	n	%	n	%	n	%	n	%	n	%	n	%
**Use of diagnostic techniques**												
Preoperative ultrasound of the axilla	54	95%	1	2%	0	0%	2	4%	0	0%	0	0%
Preoperative MRI	1	2%	9	16%	14	25%	30	53%	3	5%	0	0%
Stereotactic guidance	15	26%	35	61%	4	7%	2	4%	0	0%	1	2%
Vacuum-assisted device	11	19%	25	44%	7	12%	12	21%	1	2%	1	2%
**Decision to use SLNB is influenced by**												
National guidelines	25	44%	25	44%	4	7%	1	2%	1	2%	1	2%
Regional agreements	12	21%	19	33%	4	7%	6	11%	9	16%	7	12%
Hospital agreements	25	44%	13	23%	3	5%	5	9%	4	7%	7	12%
Multidisciplinary consultation	41	72%	12	21%	2	3%	2	3%	0	0%	0	0%
Wish of the patient	18	32%	10	18%	4	7%	21	37%	3	5%	1	2%
Own perception	8	14%	10	18%	13	23%	19	33%	4	7%	3	5%
**SLNB use for each type of surgery**												
Breast conserving surgery	3	5%	23	40%	19	33%	9	16%	3	5%	0	0%
Mastectomy	29	51%	17	30%	8	14%	3	5%	0	0%	0	0%

Perioperative ultrasound was almost always used, whereas the use of preoperative MRI, stereotactic guidance and vacuum-assisted devices was much more diverse. According to 88% of the respondents, the national guidelines always or often had an influence on the decision to use SLNB. Of the surgeons, 32% reported that wish of the patient influences the decision to use SLNB always and 37% reported that the decision on the use of SLNB is sometimes influenced by the wish of the patient. Of the surgeons, 73% reported that in their hospital SLNB was regularly or often performed in combination with breast conserving surgery, and 81% of the surgeons reported that SLNB was often or always performed in combination with mastectomy.

The associations between the reported use of SLNB and hospital factors, diagnostic techniques, and factors influencing the decision are presented in S4 File. Hospital factors like the number of patients was not associated with the use of SLNB. The techniques showing the strongest association with the use of SLNB in case of breast conserving therapy were preoperative ultrasound of the axilla and stereotactic guidance, but neither association was significant.

## Discussion

The aim of our study was to determine whether the inter-hospital variation in the use of SLNB in the Netherlands could be explained by the preferences of surgeons, organisational factors or the influence of patient wish. The preference analyses by means of BWS scenarios and ranking showed both that surgeons considered suspicion of an invasive component at biopsy and the DCIS grade to be the most important risk factors. We did not find an association between use of SLNB and organizational factors.

With the exception of ‘age’, the risk factors that surgeons indicated in this study as the most important factors in the decision to use SLNB are in agreement with the Dutch treatment guideline for patients with biopsy diagnosis of DCIS (see S5 File for the Dutch guideline). In the BWS patient scenarios, the most important factor for performing SLNB was if the patient had a suspected invasive component, and the second most important factor was the presence of DCIS grade 3. This perceived importance of high grade DCIS has previously been described in another study [9], but it is in contrast with a survey in which only 11% of the participated breast units reported to perform SLNB in case of high grade DCIS [8]. In our study, age of less than 55 years, palpability and the BI-RADS score had negative differences in the best-minus-worst case ratio, which indicates that these factors were considered the least important of the risk factors that we included in the scenarios. These results can be explained by the fact that palpability and the BI-RADS score are not included in the recommendations regarding SLNB in the Dutch DCIS treatment guideline. However, in previous research, palpability was found to be a risk factor for underestimated invasive breast cancer, which supports the inclusion of palpability in the guideline recommendation, as was done in the UK [3, 5]. In the survey study of Mannu, 65% of the surgeons agreed with that recommendation in the UK guideline, and in the study of the ‘Mammary fold academic and research collaborative’, 43% of the participated breast units reported that SLNB was done in case of mass-forming / palpable lesion [7, 8].

The BWS also indicated that there is more inter-variability between surgeons in case of BCS than in case of mastectomy. The surgeons agreed more about the most important factors for performing SLNB than about the least important factors because the a difference in the best-minus-worst ratio is closer to 1 for the most important factors than it is to -1 for the least important factors. The differences in the ratios were slightly higher for the mastectomy than for the BCS scenarios, which indicates that the surgeons agree slightly more on the most important factors for performing SLNB in case of a mastectomy.

In the ranking section of the survey, a suspected invasive component and a high DCIS grade were ranked highest. This finding strongly supports the results of the BWS, which means that there is a strong indication that the surgeons agree that these two factors constitute an indication for performing SLNB. The mammogram information, size of the lesion and a solid component were ranked as the third and fourth most important factor. Age was ranked only in eighth place, which means that the ranking and the BWS of this factor were also in accordance with each other. The respondents could mention risk factors that were missing, only MRI and wish of the patient were mentioned. This study shows that the surgeons largely agree with the Dutch guideline of 2012 regarding the risk factors defined in the recommendations for performing SLNB. The guideline itself does not describe how to deal with the combination of risk factors. Nor does it state which are the most important ones among all the risk factors nor does it indicate whether the number of risk factors that are present is most important in the decision. Perhaps the large hospital variation in the percentage of patients for whom SLNB is done is caused by this lack of decision rules for the combination of risk factors. The list of risk factors is dropped from the most recent guideline. Information on risk factors that are described in patient information which is given online is quite diverse but largely in line with the results of the ranking (see S5 File).

The results of our study indicate that the influence of the patients’ preferences is limited; in 58% to 70% of the scenarios, the surgeons reported that they would not change their decision if the patient preferred the opposite choice. In contrast, in the organisational section of the survey, 32% of the respondents stated that the decision to perform SNLB is always influenced by the patient’s preference, whereas 37% of the respondents stated that the patient’s preference only sometimes influences the decision to perform SNLB. To the best of our knowledge, the influence of the patient’s preference on the performance of SLNB has not been investigated before.

Analyses showed that the use of SLNB as reported by the surgeons was not associated with the reported background characteristics such as number of patients, nor was it associated with the reported use of diagnostic techniques such as preoperative ultrasound of the axilla, the reported influence on the decision to perform SLNB like the national guideline. While differences in hospital organisation are often actionable indicators, our results do not provide any reasons for action in this regard.

A limitation of our study’s survey regarding organisational factors was that the answers reflected the perception of the surgeons on the management in their hospitals, which means that our results might differ from the actual practice. The response rate was 22% for the BWS scenario’s and 21% for the ranking questions and therefore lower than the 30% we aimed at for that part of the questionnaire. The lower response rate might be cause by the length of the questionnaire, the response rate would have been 31% if all respondents would have completed the questionnaire. The BWS scenarios in the BWS part of the survey were balanced and nor orthogonal. For DCIS grade 1, our study produced the odd result that grade 1 was mentioned often as the most important factor for performing SLNB. Despite this issue, the answers of the surgeons confirmed that the BWS scenarios correctly indicated the order of importance of the risk factors. A strength of this study was that the same risk factors could be investigated by means of BWS scenarios and by ranking.

This study did not find any organisational factors that could explain the inter-hospital variation in the use of SLNB. Patients could have some influence on the decision. The risk factors that most surgeons reported as reasons for performing SLNB are consistent with the factors described in the Dutch treatment guideline for the use of SLNB. This means that differences in adherence to the guideline do not explain the large inter-hospital variation in the performance of SLNB, possibly this variation results from differences in dealing with the combination of risk factors.

## Supporting information

S1 File Questionnaire. (PDF)

S2 File Patient scenarios BWS. (PDF)

S3 File Decision of SLNB for the BWS scenarios. (PDF)

S4 File Background characteristics and organisational factors. (PDF)

S5 File Guidelines and hospital patient information. (PDF)
