# Supplementary material for: Surgeons’ preferences for using sentinel lymph node biopsy in patients with ductal carcinoma in situ
Source: PLoS One. 2022 Jun 6;17(6):e0269551. doi: 10.1371/journal.pone.0269551 (PMC9170095; doi:10.1371/journal.pone.0269551)
Supplement: S1 File — (PDF) [file pone.0269551.s001.pdf]

S1 Supplement: Questionnaire

Demographic Questions

The next five questions ask for some personal information. These are asked to help contextualize the answers you provide. Your answers will be processed completely anonymously.

1. What is your gender?
o Female
o Male

2. What is your specialism?
Choose one of the following options:
o General surgeon
o Oncological surgeon

3. How many years have you been working in your specialism?, as entered in the previous question.
Note: training period not included.
o 0 to 5 years
o 5 to 10 years
o 10 to 15 years
o More than 15 years

4. In which hospital do you work? …………………………………………………………………….
This question is asked in order to be able to make a decision at hospital level. During the analysis, the data is coded (the hospitals are numbered) so that your data remains anonymous but a statement can be made at hospital level.

5. How many patients with DCIS based on biopsy are treated in your hospital each year?
Choose one of the following options:
o Fewer than 10 patients per year
o 10 to 20 patients per year
o 20 to 30 patients per year
o 30 to 40 patients per year
o More than 50 patients per year

Organizational Factors

The following questions are about organizational factors regarding the sentinel lymph node biopsy.
These questions are asked to gain insight into the diagnosis and performing the sentinel lymph node biopsy for patients with biopsy-proven DCIS.
You can enter comments and apply nuances in the text box at the bottom of the page.

6. What technique do you use to find the sentinel lymph node?
o Radioactive technetium
o Patent blue
o Radioactive technetium and patent blue

7. Is there a nuclear medicine department at your hospital?
Only answer this question if you answered radioactive technetium or radioactive technetium and patent blue in the previous question.
o Yes
o No
o No, but we have a partnership with a hospital that does have this available

8. In the table below you can state whether or not you use various diagnostic tests.
Note: some questions are the situation in 2011 and some are about the situation at this moment. The variation as described is based on incidence years 2011-2012. Some diagnostic techniques may have changed in the meantime. Therefore, some questions are asked about the situation in 2011.

	Always	Often	Regularly	Sometimes	Never
I am using a pre-operative ultrasound of the axilla	○	○	○	○	○
I am using a preoperative MRI	○	○	○	○	○
I am using stereotactic biopsy	○	○	○	○	○
In 2011, I used stereotactic biopsies	○	○	○	○	○
I am using vacuum biopsies	○	○	○	○	○
In 2011, I used vacuum biopsies	○	○	○	○	○

9. In the table below you can state what influence a factor has or does not have for you in the decision to perform a sentinel lymph node biopsy.

	Influence					
	Always	Often	Regularly	Sometimes	Never	n.a.
National guideline	○	○	○	○	○	○
Regional Agreements	○	○	○	○	○	○
Hospital agreements	○	○	○	○	○	○
Advise of the multidisciplinary meeting	○	○	○	○	○	○
The wish of the patient	○	○	○	○	○	○
My own perception	○	○	○	○	○	○

10. In the table below you can state the extent to which you perform a sentinel lymph node biopsy during a mastectomy and during breast conserving surgery.

	Always	Often	Regularly	Sometimes	Never
Do you perform a sentinel lymph node biopsy on patients with biopsy-proven DCIS, undergoing mastectomy?	○	○	○	○	○
Do you perform a sentinel lymph node biopsy on patients with biopsy-proven DCIS, undergoing breast conserving surgery?	○	○	○	○	○

Room for comments and nuances for the above questions. ………………………………………………….

Scenarios

This part of the survey will consist of 16 scenarios. The scenarios consist of six patient and tumour characteristics. Some of these characteristics change with each scenario.
For each scenario, state the most important and the least important factor for you to perform a sentinel lymph node biopsy. Please note: after each scenario a question follows whether you are actually would perform a sentinel lymph node biopsy. You can select one item as the most important factor and one item as the least important factor.
We are aware that not every scenario is clinically realistic. This cannot be remedied due to the chosen methodology.
Note: a distinction has been made in the scenarios between patients undergoing breast conserving surgery and patients undergoing mastectomy. There will first be eight breast conserving surgery scenarios and then eight mastectomy scenarios.

Respondents are automatically assigned to version 1, 2, 3 or 4 of set of scenario's,
Version 1:

These first 8 scenarios are about patients undergoing breast-conserving surgery
What do you consider to be the most and least important factor in performing a sentinel lymph node procedure, given the fact that breast conserving surgery is going to take place?

<u>Scenario 1</u>
Type of surgery: breast conserving surgery

Most important factor		Least important factor
	Age 55 - 70 years	
	Not palpable	
	Size om mammogram > 2 cm	
	BIRADS score 4	
	DCIS grade 2	
	No suspicion of invasive growth on biopsy	

- Would you perform a sentinel lymph node biopsy on this patient?
  - Yes
  - No
- Suppose the patient prefers the opposite to your answer above, would this change your decision?
  - Yes
  - No

<u>Scenario 2</u>
Type of surgery: breast conserving surgery

Most important factor		Least important factor
	Age between 55 and 70 years	
	Not palpable	
	Size on mammogram < = 2 cm	
	BIRADS score 4	
	DCIS grade 1	
	No suspicion of invasive growth on biopsy	

- Would you perform a sentinel lymph node biopsy on this patient?
  - Yes
  - No
- Suppose the patient prefers the opposite to your answer above, would this change your decision?

- Yes
- No

Scenario 3

Type of surgery: breast conserving surgery

Most important factor		Least important factor
	Age > 70 years	
	Palpable	
	Size on mammogram <= 2 cm	
	BIRADS score 5	
	DCIS grade 3	
	No suspicion of invasive growth on biopsy	

- Would you perform a sentinel lymph node biopsy on this patient?
  - Yes
  - No
- Suppose the patient prefers the opposite to your answer above, would this change your decision?
  - Yes
  - No

Scenario 4

Type of surgery: breast conserving surgery

Most important factor		Least important factor
	Age > 70 years	
	Not palpable	
	Size on mammogram <= to 2 cm	
	BIRADS score 5	
	DCIS grade 1	
	No suspicion of invasive growth on biopsy	

- Would you perform a sentinel lymph node biopsy on this patient?
  - Yes
  - No
- Suppose the patient prefers the opposite to your answer above, would this change your decision?
  - Yes
  - No

Scenario 5

Type of surgery: breast conserving surgery

Most important factor		Least important factor
	Age < 55 years	
	Palpable	
	Size on mammogram > 2 cm	
	BIRADS score 4	
	DCIS grade 3	
	Suspected invasive growth on biopsy	

- Would you perform a sentinel lymph node biopsy on this patient?
  - Yes
  - No
- Suppose the patient prefers the opposite to your answer above, would this change your decision?
  - Yes

- No

Scenario 6

Type of surgery: breast conserving surgery

Most important factor		Least important factor
	Age < 55 years	
	Not palpable	
	Size on mammogram > 2 cm	
	BIRADS score 5	
	DCIS grade 2	
	Suspected invasive growth on biopsy	

- Would you perform a sentinel lymph node biopsy on this patient?
  - Yes
  - No
- Suppose the patient prefers the opposite to your answer above, would this change your decision?
  - Yes
  - No

Scenario 7

Type of surgery: breast conserving surgery

Most important factor		Least important factor
	Age 55 - 70 years	
	Palpable	
	Size on mammogram <= 2 cm	
	BIRADS score 4	
	DCIS grade 1	
	Suspected invasive growth on biopsy	

- Would you perform a sentinel lymph node biopsy on this patient?
  - Yes
  - No
- Suppose the patient prefers the opposite to your answer above, would this change your decision?
  - Yes
  - No

Scenario 8

Type of surgery: breast conserving surgery

Most important factor		Least important factor
	Age < 55 years	
	Palpable	
	Size on mammogram > 2 cm	
	BIRADS score 5	
	DCIS grade 2	
	Suspected invasive growth on biopsy	

- Would you perform a sentinel lymph node biopsy on this patient?
  - Yes
  - No
- Suppose the patient prefers the opposite to your answer above, would this change your decision?
  - Yes
  - No

The next eight scenarios will be about patients undergoing a mastectomy. For each scenario, state the most important and the least important factor for you to perform a sentinel lymph node biopsy given that a mastectomy is performed.
Please note: after each scenario a question follows whether you are actually would perform a sentinel lymph node biopsy. You can select one item as the most important factor and one item as the least important factor.
We are aware that not every scenario is clinically realistic. This cannot be remedied due to the chosen methodology.

The following eight scenarios are about patients undergoing a mastectomy.
At the end of the eight scenarios, one ranking question is asked. The intention is that you rank the various factors that influence the decision on performing the sentinel lymph node biopsy.

<u>Scenario 9</u>
Type of surgery: mastectomy

Most important factor		Least important factor
	Age 55 - 70 years	
	Not palpable	
	Size om mammogram > 2 cm	
	BIRADS score 4	
	DCIS grade 2	
	No suspicion of invasive growth on biopsy	

- Would you perform a sentinel lymph node biopsy on this patient?
    - Yes
    - No
- Suppose the patient prefers the opposite to your answer above, would this change your decision?
    - Yes
    - No

<u>Scenario 10</u>
Type of surgery: mastectomy

Most important factor		Least important factor
	Age between 55 and 70 years	
	Not palpable	
	Size on mammogram < = 2 cm	
	BIRADS score 4	
	DCIS grade 1	
	No suspicion of invasive growth on biopsy	

- Would you perform a sentinel lymph node biopsy on this patient?
    - Yes
    - No
- Suppose the patient prefers the opposite to your answer above, would this change your decision?
    - Yes
    - No

<u>Scenario 11</u>
Type of surgery: mastectomy

Most important factor		Least important factor
	Age > 70 years	
	Palpable	

	Size on mammogram <= 2 cm	
	BIRADS score 5	
	DCIS grade 3	
	No suspicion of invasive growth on biopsy	

- Would you perform a sentinel lymph node biopsy on this patient?
  - Yes
  - No
- Suppose the patient prefers the opposite to your answer above, would this change your decision?
  - Yes
  - No

Scenario 12

Type of surgery: mastectomy

Most important factor		Least important factor
	Age > 70 years	
	Not palpable	
	Size on mammogram <= to 2 cm	
	BIRADS score 5	
	DCIS grade 1	
	No suspicion of invasive growth on biopsy	

- Would you perform a sentinel lymph node biopsy on this patient?
  - Yes
  - No
- Suppose the patient prefers the opposite to your answer above, would this change your decision?
  - Yes
  - No

Scenario 13

Type of surgery: mastectomy

Most important factor		Least important factor
	Age < 55 years	
	Palpable	
	Size on mammogram > 2 cm	
	BIRADS score 4	
	DCIS grade 3	
	Suspected invasive growth on biopsy	

- Would you perform a sentinel lymph node biopsy on this patient?
  - Yes
  - No
- Suppose the patient prefers the opposite to your answer above, would this change your decision?
  - Yes
  - No

Scenario 14

Type of surgery: mastectomy

Most important factor		Least important factor
	Age < 55 years	
	Not palpable	
	Size on mammogram > 2 cm	

	BIRADS score 5	
	DCIS grade 2	
	Suspected invasive growth on biopsy	

- Would you perform a sentinel lymph node biopsy on this patient?
  - Yes
  - No
- Suppose the patient prefers the opposite to your answer above, would this change your decision?
  - Yes
  - No

<u>Scenario 15</u>

Type of surgery: mastectomy

Most important factor		Least important factor
	Age 55 - 70 years	
	Palpable	
	Size on mammogram <= 2 cm	
	BIRADS score 4	
	DCIS grade 1	
	Suspected invasive growth on biopsy	

- Would you perform a sentinel lymph node biopsy on this patient?
  - Yes
  - No
- Suppose the patient prefers the opposite to your answer above, would this change your decision?
  - Yes
  - No

<u>Scenario 16</u>

Type of surgery: mastectomy

Most important factor		Least important factor
	Age < 55 years	
	Palpable	
	Size on mammogram > 2 cm	
	BIRADS score 5	
	DCIS grade 2	
	Suspected invasive growth on biopsy	

- Would you perform a sentinel lymph node biopsy on this patient?
  - Yes
  - No
- Suppose the patient prefers the opposite to your answer above, would this change your decision?
  - Yes
  - No

11. Below are 12 factors that can influence the decision whether or not to perform sentinel lymph node biopsy. You will be asked to rank the factors by importance. The answers must be different and must be ranked. Determine the sequence number from 1 to 12 for each factor.

Ranking number	Factors
	Age
	DCIS grade
	Size on mammography
	Yes/no suspicion of invasive growth on biopsy
	BIRADS score
	Yes/no direct breast reconstruction
	Palpable/not palpable
	Yes/no found by population screening
	Type of surgery (breast conserving surgery / mastectomy)
	Multifocality/multicentricity
	Yes/no contralateral tumour
	Yes /no solid component on mammography

- If you are missing factors in the previous question that you think influences the decision on performing sentinel node lymph node biopsy, you can state the missing factors here………………………………………………………

End of questionnaire

Thank you for completing this survey. If you are interested in the results of this survey, you can leave your email address here……………………………

If you have any comments about the survey, the guideline or the use of sentinel lymph node biopsy, you can state them here………………………………..

Thank you for participating in this survey.

Vragenlijst

Demografische vragen

In de komende vijf vragen zal er gevraagd worden naar enkele persoonlijke gegevens. Deze vragen worden gesteld om de door u gegeven antwoorden in een context te kunnen plaatsen. Uw antwoorden worden volledig anoniem verwerkt.

1. Wat is uw geslacht?

Kies één van de volgende mogelijkheden:
- o Vrouw
- o Man

2. Wat is uw specialisme?

Kies één van de volgende mogelijkheden:
- o Algemeen chirurg
- o Oncologisch chirurg

3. Hoeveel jaar bent u werkzaam in uw specialisme? Zoals ingevuld bij de vorige vraag. Let op: opleidingsperiode niet meegeteld.

Kies één van de volgende mogelijkheden:
- o 0 tot 5 jaar
- o 5 tot 10 jaar
- o 10 tot 15 jaar
- o Meer dan 15 jaar

4. In welk ziekenhuis bent u werkzaam? *

Deze vraag wordt gesteld om uitspraak te kunnen doen op ziekenhuisniveau. Tijdens de analyse worden de gegevens gecodeerd (de ziekenhuizen genummerd) zodat uw gegevens anoniem blijven maar er wel uitspraak gedaan kan worden op ziekenhuis niveau.

Vul uw antwoord hier in:

5. Hoeveel patiënten met DCIS op basis van biopsie, worden jaarlijks in uw ziekenhuis behandeld?

Kies één van de volgende mogelijkheden:
- o Minder dan 10 patiënten per jaar
- o 10 tot 20 patiënten per jaar
- o 20 tot 30 patiënten per jaar
- o 30 tot 40 patiënten per jaar
- o Meer dan 50 patiënten per jaar

Organisatorische factoren

De komende vragen gaan over organisatorische factoren rondom het uitvoeren van de schildwachtklier procedure. Deze vragen worden gesteld om de gang van zaken rondom de diagnose en het uitvoeren van de schildwachtklier procedure bij patiënten met DCIS in kaart te brengen. Opmerkingen en nuances kunt u onderaan de pagina in het tekstvlak kwijt.

6. Welke techniek gebruikt u voor het identificeren van de schildwachtklier? * Kies één van de

volgende mogelijkheden:

- o Radioactief technetium
- o Patent blue
- o Radioactief technetium en patent blue

7. Is er binnen uw ziekenhuis een afdeling nucleaire geneeskunde tot uw beschikking? Beantwoord deze vraag alleen als u bij de vorige vraag radioactief technetium of radioactief technetium en patent blue als antwoord hebt gegeven.

Kies één van de volgende mogelijkheden:

- o Ja
- o Nee
- o Nee, maar wij hebben een samenwerkingsverband met een ziekenhuis die hier wel over beschikt

8. In onderstaande tabel kunt u aanklikken in hoeverre u al dan niet gebruik maakt van diverse diagnosticerende onderzoeken. Let op: enkele vragen gaan over het gebruik in 2011 en enkele vragen gaan over het gebruik op dit moment.

De variatie zoals deze beschreven is, is gebaseerd op incidentiejaren 2011-2012. Mogelijk zijn een aantal diagnosticerende onderzoeken in de tussentijd gewijzigd. Daarom wordt voor enkele onderzoeken apart gevraagd naar de situatie in 2011.

	Altijd	Vaak	Regelmatig	Soms	Nooit
Ik maak op dit moment gebruik van een preoperatieve echo van de oksel bij patiënten met DCIS	○	○	○	○	○
Ik maak op dit moment gebruik van een preoperatieve MRI van de afwijking bij patiënten met DCIS	○	○	○	○	○
Ik maak op dit moment gebruik van stereotactische biopsieën bij patiënten met DCIS.	○	○	○	○	○
In 2011 heb ik gebruik gemaakt van stereotactische biopsieën bij patiënten met DCIS	○	○	○	○	○
Ik maak op dit moment gebruik van vacuüm biopsieën bij patiënten met DCIS	○	○	○	○	○
In 2011 heb ik gebruik gemaakt van vacuüm biopsieën bij patiënten met DCIS	○	○	○	○	○

9. In onderstaande tabel kunt u aangeven welke invloed een factor al dan niet heeft voor u bij de beslissing om een schildwachtklier procedure uit te voeren.

Kies het toepasselijke antwoord voor elk onderdeel:

	Altijd invloed	Vaak invloed	Regelmatig invloed	Soms invloed	Geen invloed	Niet van toepassing
Landelijke richtlijn	o	o	o	o	o	o
Regionale afspraken	o	o	o	o	o	o
Ziekenhuis afspraken	o	o	o	o	o	o
Het MDO advies	o	o	o	o	o	o
De wens van de patiënt	o	o	o	o	o	o
Mijn eigen perceptie	o	o	o	o	o	o

10. In onderstaande tabel kunt u aanklikken in hoeverre u een schildwachtklier procedure uitvoert bij een mastectomie en een borstsparende operatie.

Kies het toepasselijke antwoord voor elk onderdeel:

	Altijd	Vaak	Regelmatig	Soms	Nooit
Voert u een schildwachtklier procedure uit bij patiënten met DCIS die een mastectomie ondergaan?	o	o	o	o	o
Voert u een schildwachtklier procedure uit bij patiënten met DCIS die een borstsparende operatie ondergaan?	o	o	o	o	o

Ruimte voor opmerkingen en nuances voor bovenstaande vragen. Vul uw antwoord hier in:

Scenarios

Dit onderdeel van de enquête zal bestaan uit 16 scenario's waarin een beeld geschetst wordt van patiënten met DCIS. De scenario's bestaan uit een zestal patiënt- en tumorkenmerken. Een aantal van deze kenmerken veranderen bij elk scenario.

Bij elk scenario is het de bedoeling dat u aangeeft wat voor u de meest belangrijke en de minst belangrijke factor is om wél een schildwachtklier procedure uit te voeren. Let op: na elk scenario volgt een vraag of u een schildwachtklier procedure dan ook daadwerkelijk uitvoert. U kunt één item aanklikken als meest belangrijke factor en één item als minst belangrijke factor.

Wij zijn er ons van bewust dat niet elk scenario klinisch gezien even realistisch is. Dit is wegens de gekozen methodologie niet te verhelpen.

Let op: er is onderscheid gemaakt in de scenario's tussen patiënten die een borstsparende operatie ondergaan en patiënten met een mastectomie. Er zullen eerst 8 borstsparende scenario's aan bod komen en vervolgens 8 mastectomie scenario's.

Deze eerste 8 scenario's gaan over patiënten die een borstsparende operatie ondergaan

Wat vindt u de meest en minst belangrijke factor om een schildwachtklier procedure uit te voeren, gegeven het feit dat er een borstsparende operatie gaat plaatsvinden.

Scenario 1

Type operatie: borstsparende operatie

Meest belangrijke factor		Minst belangrijke factor
	Leeftijd tussen 55 tot 70 jaar	
	Niet palpabel	
	Afmeting afwijking mammogram groter dan 2 cm	
	BIRAD score 4	
	DCIS graad 2	
	Geen verdenking invasieve groei op biopsie	

- Zou u bij deze patiënt een schildwachtklier procedure uitvoeren?

* Kies één van de volgende mogelijkheden:

- o Ja
- o Nee

- Stel de patiënt heeft de voorkeur, tegenovergesteld aan uw antwoord van zojuist. Zou dit uw keuze van zojuist veranderen?

Kies één van de volgende mogelijkheden:

- o Ja
- o Nee

<u>Scenario 2</u>

Type operatie: borstsparende operatie

Meest belangrijke factor		Minst belangrijke factor
	Leeftijd tussen 55 tot 70 jaar	
	Niet palpabel	
	Afmeting afwijking mammogram kleiner of gelijk aan 2 cm	
	BIRAD score 4	
	DCIS graad 1	
	Geen verdenking invasieve groei op biopsie	

➢ Zou u bij deze patiënt een schildwachtklier procedure uitvoeren? * Kies één van de volgende mogelijkheden:

- o Ja
- o Nee

➢ Stel de patiënt heeft de voorkeur, tegenovergesteld aan uw antwoord van zojuist. Zou dit uw keuze van zojuist veranderen?

Kies één van de volgende mogelijkheden:

- o Ja
- o Nee

<u>Scenario 3</u>

Type operatie: borstsparende operatie

Meest belangrijke factor		Minst belangrijke factor
	Leeftijd ouder dan 70 jaar	
	Palpabel	
	Afmeting afwijking mammogram kleiner of gelijk aan 2 cm	
	BIRAD score 5	
	DCIS graad 3	
	Geen verdenking invasieve groei op biopsie	

➢ Zou u bij deze patiënt een schildwachtklier procedure uitvoeren? * Kies één van de volgende mogelijkheden:

- o Ja
- o Nee

➢ Stel de patiënt heeft de voorkeur, tegenovergesteld aan uw antwoord van zojuist. Zou dit uw keuze van zojuist veranderen?

Kies één van de volgende mogelijkheden:

- o Ja
- o Nee

<u>Scenario 4</u>

Type operatie: borstsparende operatie

Meest belangrijke factor		Minst belangrijke factor
	Leeftijd ouder dan 70 jaar	
	Niet palpabel	
	Afmeting afwijking mammogram kleiner of gelijk aan 2 cm	
	BIRAD score 5	

	DCIS graad 1	
	Geen verdenking invasieve groei op biopsie	

- Zou u bij deze patiënt een schildwachtklier procedure uitvoeren? * Kies één van de volgende mogelijkheden:
  - Ja
  - Nee
- Stel de patiënt heeft de voorkeur, tegenovergesteld aan uw antwoord van zojuist. Zou dit uw keuze van zojuist veranderen?
  Kies één van de volgende mogelijkheden:
  - Ja
  - Nee

<u>Scenario 5</u>

Type operatie: borstsparende operatie

Meest belangrijke factor		Minst belangrijke factor
	Leeftijd jonger dan 55 jaar	
	Palpabel	
	Afmeting afwijking mammogram groter dan 2 cm	
	BIRAD score 4	
	DCIS graad 3	
	Verdenking invasieve groei op biopsie	

- Zou u bij deze patiënt een schildwachtklier procedure uitvoeren? * Kies één van de volgende mogelijkheden:
  - Ja
  - Nee
- Stel de patiënt heeft de voorkeur, tegenovergesteld aan uw antwoord van zojuist. Zou dit uw keuze van zojuist veranderen?

Kies één van de volgende mogelijkheden:
  - Ja
  - Nee

<u>Scenario 6</u>

Type operatie: borstsparende operatie

Meest belangrijke factor		Minst belangrijke factor
	Leeftijd jonger dan 55 jaar	
	Niet palpabel	
	Afmeting afwijking mammogram groter dan 2 cm	
	BIRAD score 5	
	DCIS graad 2	
	Verdenking invasieve groei op biopsie	

- Zou u bij deze patiënt een schildwachtklier procedure uitvoeren? * Kies één van de volgende mogelijkheden:
  - Ja
  - Nee
- Stel de patiënt heeft de voorkeur, tegenovergesteld aan uw antwoord van zojuist. Zou dit uw keuze van zojuist veranderen?

Kies één van de volgende mogelijkheden:
  - Ja
  - Nee

<u>Scenario 7</u>

Type operatie: borstsparende operatie

Meest belangrijke factor		Minst belangrijke factor
	Leeftijd tussen 55 en 70 jaar	
	Palpabel	
	Afmeting afwijking mammogram kleiner of gelijk aan 2 cm	
	BIRAD score 4	
	DCIS graad 1	
	Verdenking invasieve groei op biopsie	

➢ Zou u bij deze patiënt een schildwachtklier procedure uitvoeren? * Kies één van de volgende mogelijkheden:

- Ja
- Nee

➢ Stel de patiënt heeft de voorkeur, tegenovergesteld aan uw antwoord van zojuist. Zou dit uw keuze van zojuist veranderen?

Kies één van de volgende mogelijkheden:

- Ja
- Nee

Scenario 8

Type operatie: borstsparende operatie

Meest belangrijke factor		Minst belangrijke factor
	Leeftijd jonger dan 55 jaar	
	Palpabel	
	Afmeting afwijking mammogram groter dan 2 cm	
	BIRAD score 5	
	DCIS graad 2	
	Verdenking invasieve groei op biopsie	

➢ Zou u bij deze patiënt een schildwachtklier procedure uitvoeren? * Kies één van de volgende mogelijkheden:

- Ja
- Nee

➢ Stel de patiënt heeft de voorkeur, tegenovergesteld aan uw antwoord van zojuist. Zou dit uw keuze van zojuist veranderen?

Kies één van de volgende mogelijkheden:

- Ja
- Nee

De komende 8 scenario's zullen gaan over patiënten die een mastectomie ondergaan. Wat vindt u in een bepaald scenario de meest en minst belangrijke factor om een schildwachtklier procedure uit te voeren, gegeven het feit dat er een mastectomie plaats gaat vinden.
Let op: na elk scenario volgt een vraag of u een schildwachtklier procedure dan ook daadwerkelijk uitvoert. U kunt één item aanklikken als meest belangrijke factor en één item als minst belangrijke factor.
Wij zijn er ons van bewust dat niet elk scenario klinisch gezien even realistisch is. Dit is wegens de gekozen methodologie niet te verhelpen.

Deze 8 scenario's gaan over patiënten die een mastectomie ondergaan

Na afloop van de 8 scenario's wordt één ranking vraag gesteld. Hierbij is het de bedoeling dat u de verschillende factoren die van invloed zijn bij de keuze voor een schildwachtklier procedure rankt.

Scenario 9

Type operatie: mastectomie

Meest belangrijke factor		Minst belangrijke factor
	Leeftijd tussen 55 tot 70 jaar	
	Niet palpabel	
	Afmeting afwijking mammogram groter dan 2 cm	
	BIRAD score 4	
	DCIS graad 2	
	Geen verdenking invasieve groei op biopsie	

- Zou u bij deze patiënt een schildwachtklier procedure uitvoeren? * Kies één van de volgende mogelijkheden:
  - Ja
  - Nee
- Stel de patiënt heeft de voorkeur, tegenovergesteld aan uw antwoord van zojuist. Zou dit uw keuze van zojuist veranderen?

Kies één van de volgende mogelijkheden:
  - Ja
  - Nee

Scenario 10

Type operatie: mastectomie

Meest belangrijke factor		Minst belangrijke factor
	Leeftijd tussen 55 tot 70 jaar	
	Niet palpabel	
	Afmeting afwijking mammogram kleiner of gelijk aan 2 cm	
	BIRAD score 4	
	DCIS graad 1	
	Geen verdenking invasieve groei op biopsie	

- Zou u bij deze patiënt een schildwachtklier procedure uitvoeren? * Kies één van de volgende mogelijkheden:
  - Ja
  - Nee
- Stel de patiënt heeft de voorkeur, tegenovergesteld aan uw antwoord van zojuist. Zou dit uw keuze van zojuist veranderen?

Kies één van de volgende mogelijkheden:
  - Ja
  - Nee

Scenario 11

Type operatie: mastectomie

Meest belangrijke factor		Minst belangrijke factor
	Leeftijd ouder dan 70 jaar	
	Palpabel	
	Afmeting afwijking mammogram kleiner of gelijk aan 2 cm	
	BIRAD score 5	
	DCIS graad 3	
	Geen verdenking invasieve groei op biopsie	

- Zou u bij deze patiënt een schildwachtklier procedure uitvoeren? * Kies één van de volgende mogelijkheden:
  - Ja

- Nee

- Stel de patiënt heeft de voorkeur, tegenovergesteld aan uw antwoord van zojuist. Zou dit uw keuze van zojuist veranderen?

Kies één van de volgende mogelijkheden:

- Ja
- Nee

Scenario 12

Type operatie: mastectomie

Meest belangrijke factor		Minst belangrijke factor
	Leeftijd ouder dan 70 jaar	
	Niet palpabel	
	Afmeting afwijking mammogram kleiner of gelijk aan 2 cm	
	BIRAD score 5	
	DCIS graad 1	
	Geen verdenking invasieve groei op biopsie	

- Zou u bij deze patiënt een schildwachtklier procedure uitvoeren? * Kies één van de volgende mogelijkheden:

- Ja
- Nee

- Stel de patiënt heeft de voorkeur, tegenovergesteld aan uw antwoord van zojuist. Zou dit uw keuze van zojuist veranderen?

Kies één van de volgende mogelijkheden:

- Ja
- Nee

Scenario 13

Type operatie: mastectomie

Meest belangrijke factor		Minst belangrijke factor
	Leeftijd jonger dan 55 jaar	
	Palpabel	
	Afmeting afwijking mammogram groter dan 2 cm	
	BIRAD score 4	
	DCIS graad 3	
	Verdenking invasieve groei op biopsie	

- Zou u bij deze patiënt een schildwachtklier procedure uitvoeren? * Kies één van de volgende mogelijkheden:

- Ja
- Nee

- Stel de patiënt heeft de voorkeur, tegenovergesteld aan uw antwoord van zojuist. Zou dit uw keuze van zojuist veranderen?

Kies één van de volgende mogelijkheden:

- Ja
- Nee

Scenario 14

Type operatie: mastectomie

Meest belangrijke factor		Minst belangrijke factor
	Leeftijd jonger dan 55 jaar	
	Niet palpabel	

	Afmeting afwijking mammogram groter dan 2 cm	
	BIRAD score 5	
	DCIS graad 2	
	Verdenking invasieve groei op biopsie	

- ➢ Zou u bij deze patiënt een schildwachtklier procedure uitvoeren? * Kies één van de volgende mogelijkheden:
  - o Ja
  - o Nee
- ➢ Stel de patiënt heeft de voorkeur, tegenovergesteld aan uw antwoord van zojuist. Zou dit uw keuze van zojuist veranderen?

Kies één van de volgende mogelijkheden:
  - o Ja
  - o Nee

<u>Scenario 15</u>

Type operatie: mastectomie

Meest belangrijke factor		Minst belangrijke factor
	Leeftijd tussen 55 en 70 jaar	
	Palpabel	
	Afmeting afwijking mammogram kleiner of gelijk aan 2 cm	
	BIRAD score 4	
	DCIS graad 1	
	Verdenking invasieve groei op biopsie	

- ➢ Zou u bij deze patiënt een schildwachtklier procedure uitvoeren? * Kies één van de volgende mogelijkheden:
  - o Ja
  - o Nee
- ➢ Stel de patiënt heeft de voorkeur, tegenovergesteld aan uw antwoord van zojuist. Zou dit uw keuze van zojuist veranderen?

Kies één van de volgende mogelijkheden:
  - o Ja
  - o Nee

<u>Scenario 16</u>

Type operatie: mastectomie

Meest belangrijke factor		Minst belangrijke factor
	Leeftijd jonger dan 55 jaar	
	Palpabel	
	Afmeting afwijking mammogram groter dan 2 cm	
	BIRAD score 5	
	DCIS graad 2	
	Verdenking invasieve groei op biopsie	

- ➢ Zou u bij deze patiënt een schildwachtklier procedure uitvoeren? * Kies één van de volgende mogelijkheden:
  - o Ja
  - o Nee
- ➢ Stel de patiënt heeft de voorkeur, tegenovergesteld aan uw antwoord van zojuist. Zou dit uw keuze van zojuist veranderen?

Kies één van de volgende mogelijkheden:
  - o Ja
  - o Nee

11. Hieronder staan 12 factoren die invloed kunnen hebben op de beslissing wel of geen schildwachtklier procedure. U wordt gevraagd de factoren op belangrijkheid te ranken.
De antwoorden moeten verschillend zijn en moeten worden gerangschikt. Bepaal voor elke optie het volgnummer van 1 tot 12

Ranking nummer	Factoren
	Leeftijd patiënt
	Graad DCIS
	Afmeting afwijking mammogram
	Wel/geen verdenking invasieve groei op biopsie
	BIRAD score
	Wel/geen directe reconstructie
	Palpabel/niet palpabel
	Afwijking wel/niet gevonden tijdens bevolkingsonderzoek
	Type operatie(borstsparend/mastectomie)
	Multifocaliteit/multicentriciteit
	Wel/geen contralaterale tumor
	Wel/geen solide component op mammografie

- Indien u bij de vorige vraag factoren mist die volgens u van invloed zijn op de keuze rondom een schildwachtklier procedure dan kunt u hier de missende factoren vermelden.

Einde vragenlijst

Hartelijk dank voor het invullen van deze enquête. Indien u geïnteresseerd bent in de uitkomsten van dit onderzoek dan kunt u hieronder uw e-mailadres achterlaten.

Indien u opmerkingen heeft over de enquête, de richtlijn of het toepassen van de schildwachtklier procedure, dan kunt u dit hieronder vermelden.

Bedankt voor uw deelname aan deze enquête.
