# Supplementary material for: Surgeons’ preferences for using sentinel lymph node biopsy in patients with ductal carcinoma in situ
Source: PLoS One. 2022 Jun 6;17(6):e0269551. doi: 10.1371/journal.pone.0269551 (PMC9170095; doi:10.1371/journal.pone.0269551)
Supplement: S2 File — (PDF) [file pone.0269551.s002.pdf]

# S2 supplement: patient scenarios BWS

Level of each attribute as included in the BWS patient scenarios.

| Version | Scenario | Age in years | Palpable | Size on mammogram | BI-RADS score | DCIS grade | Suspected invasive component |
|---|---|---|---|---|---|---|---|
| 1 | 1 | 55 - 70 | No | > 2cm | Score 4 | Grade 2 | No |
| 1 | 2 | 55 - 70 | No | ≤ 2 cm | Score 4 | Grade 1 | No |
| 1 | 3 | >70 | Yes | ≤ 2 cm | Score 5 | Grade 3 | No |
| 1 | 4 | >70 | No | ≤ 2 cm | Score 5 | Grade 1 | No |
| 1 | 5 | < 55 | Yes | > 2cm | Score 4 | Grade 3 | Yes |
| 1 | 6 | < 55 | No | > 2cm | Score 5 | Grade 2 | Yes |
| 1 | 7 | 55 - 70 | Yes | ≤ 2 cm | Score 4 | Grade 1 | Yes |
| 1 | 8 | < 55 | Yes | > 2cm | Score 5 | Grade 2 | Yes |
| 2 | 1 | >70 | No | ≤ 2 cm | Score 4 | Grade 2 | No |
| 2 | 2 | < 55 | Yes | ≤ 2 cm | Score 5 | Grade 3 | No |
| 2 | 3 | 55 - 70 | Yes | > 2cm | Score 5 | Grade 2 | Yes |
| 2 | 4 | >70 | No | > 2cm | Score 4 | Grade 1 | No |
| 2 | 5 | < 55 | No | ≤ 2 cm | Score 4 | Grade 3 | No |
| 2 | 6 | < 55 | No | ≤ 2 cm | Score 5 | Grade 2 | Yes |
| 2 | 7 | >70 | Yes | ≤ 2 cm | Score 4 | Grade 3 | Yes |
| 2 | 8 | 55 - 70 | Yes | > 2cm | Score 5 | Grade 3 | Yes |
| 3 | 1 | < 55 | Yes | ≤ 2 cm | Score 5 | Grade 2 | Yes |
| 3 | 2 | 55 - 70 | Yes | ≤ 2 cm | Score 5 | Grade 2 | No |
| 3 | 3 | 55 - 70 | No | ≤ 2 cm | Score 4 | Grade 3 | No |
| 3 | 4 | < 55 | No | ≤ 2 cm | Score 4 | Grade 1 | No |
| 3 | 5 | >70 | Yes | > 2cm | Score 4 | Grade 1 | No |
| 3 | 6 | < 55 | Yes | > 2cm | Score 5 | Grade 3 | Yes |
| 3 | 7 | >70 | No | > 2cm | Score 4 | Grade 1 | Yes |
| 3 | 8 | >70 | No | > 2cm | Score 5 | Grade 2 | Yes |
| 4 | 1 | >70 | Yes | ≤ 2 cm | Score 5 | Grade 3 | No |
| 4 | 2 | 55 - 70 | No | > 2cm | Score 5 | Grade 3 | No |
| 4 | 3 | 55 - 70 | No | > 2cm | Score 5 | Grade 2 | Yes |
| 4 | 4 | < 55 | Yes | > 2cm | Score 5 | Grade 3 | No |
| 4 | 5 | 55 - 70 | No | ≤ 2 cm | Score 4 | Grade 1 | Yes |
| 4 | 6 | < 55 | Yes | > 2cm | Score 4 | Grade 2 | Yes |
| 4 | 7 | >70 | Yes | ≤ 2 cm | Score 4 | Grade 1 | No |
| 4 | 8 | 55 - 70 | Yes | > 2cm | Score 4 | Grade 1 | Yes |
