# Supplementary material for: Surgeons’ preferences for using sentinel lymph node biopsy in patients with ductal carcinoma in situ
Source: PLoS One. 2022 Jun 6;17(6):e0269551. doi: 10.1371/journal.pone.0269551 (PMC9170095; doi:10.1371/journal.pone.0269551)
Supplement: S3 File — (PDF) [file pone.0269551.s003.pdf]

S3 supplement: decision of SLNB for the BWS scenarios

For each scenario the following is given:

- total number of respondent that answered the question
- number of respondents that would perform the SLNB
- number of respondents that would still perform the SLNB if the patient wished the opposite

Scenarios that are clinically interesting are highlighted in red.

Version	Scenario	Age in years	Palpable	Size on mammogram	BI-RADS score	DCIS grade	Suspected inv. component	Total number respondents	SLNB: Yes	Still Yes, wish No	Total	SLNB: Yes	Still Yes, wish No
								BCS			Mastectomy		
1	1	55 - 70	No	> 2cm	Score 4	Grade 2	No	12	7	3	12	9	6
1	2	55 - 70	No	≤ 2 cm	Score 4	Grade 1	No	12	0	n.a.	12	5	2
1	3	>70	Yes	≤ 2 cm	Score 5	Grade 3	No	12	12	7	12	10	7
1	4	>70	No	≤ 2 cm	Score 5	Grade 1	No	12	2	1	12	6	1
1	5	< 55	Yes	> 2cm	Score 4	Grade 3	Yes	12	12	11	12	12	11
1	6	< 55	No	> 2cm	Score 5	Grade 2	Yes	12	12	11	12	12	11
1	7	55 - 70	Yes	≤ 2 cm	Score 4	Grade 1	Yes	12	8	5	12	10	5
1	8	< 55	Yes	> 2cm	Score 5	Grade 2	Yes	12	12	11	12	12	11
2	1	>70	No	≤ 2 cm	Score 4	Grade 2	No	14	6	0	14	13	5
2	2	< 55	Yes	≤ 2 cm	Score 5	Grade 3	No	14	12	5	14	14	10
2	3	55 - 70	Yes	> 2cm	Score 5	Grade 2	Yes	14	14	8	14	14	12
2	4	>70	No	> 2cm	Score 4	Grade 1	No	14	1	0	14	6	2
2	5	< 55	No	≤ 2 cm	Score 4	Grade 3	No	14	11	4	14	14	12
2	6	< 55	No	≤ 2 cm	Score 5	Grade 2	Yes	14	14	10	14	14	12
2	7	>70	Yes	≤ 2 cm	Score 4	Grade 3	Yes	14	14	10	14	14	12
2	8	55 - 70	Yes	> 2cm	Score 5	Grade 3	Yes	14	14	12	14	14	12
3	1	< 55	Yes	≤ 2 cm	Score 5	Grade 2	Yes	18	18	11	18	18	14
3	2	55 - 70	Yes	≤ 2 cm	Score 5	Grade 2	No	18	13	6	18	15	8
3	3	55 - 70	No	≤ 2 cm	Score 4	Grade 3	No	18	15	5	18	18	12
3	4	< 55	No	≤ 2 cm	Score 4	Grade 1	No	18	0	n.a.	18	8	3
3	5	>70	Yes	> 2cm	Score 4	Grade 1	No	18	6	0	18	15	4
3	6	< 55	Yes	> 2cm	Score 5	Grade 3	Yes	18	18	14	18	18	15
3	7	>70	No	> 2cm	Score 4	Grade 1	Yes	18	15	6	18	17	11
3	8	>70	No	> 2cm	Score 5	Grade 2	Yes	18	18	8	18	18	12
4	1	>70	Yes	≤ 2 cm	Score 5	Grade 3	No	13	12	8	13	12	8
4	2	55 - 70	No	> 2cm	Score 5	Grade 3	No	13	13	9	13	13	9
4	3	55 - 70	No	> 2cm	Score 5	Grade 2	Yes	13	13	8	13	13	10
4	4	< 55	Yes	> 2cm	Score 5	Grade 3	No	13	13	10	13	13	10
4	5	55 - 70	No	≤ 2 cm	Score 4	Grade 1	Yes	13	10	3	13	11	5
4	6	< 55	Yes	> 2cm	Score 4	Grade 2	Yes	13	10	5	13	10	8
4	7	>70	Yes	≤ 2 cm	Score 4	Grade 1	No	13	4	1	13	5	3
4	8	55 - 70	Yes	> 2cm	Score 4	Grade 1	Yes	13	11	7	13	12	8
