# Supplementary material for: Surgeons’ preferences for using sentinel lymph node biopsy in patients with ductal carcinoma in situ
Source: PLoS One. 2022 Jun 6;17(6):e0269551. doi: 10.1371/journal.pone.0269551 (PMC9170095; doi:10.1371/journal.pone.0269551)

S4 supplement: background characteristics and organisational factors

Associations between background characteristics and organisational factors and the reported use of SLNB for breast conserving surgery and for mastectomy.

		Fisher exact test (p-value)	
		**BCS**	**Mastectomy**
**Hospital**			
	Number of patients with DCIS each year in your hospital	0.984	0.883
	Technique used to identify sentinel lymph node	0.608	0.108
	Availability of nuclear medicine	0.896	0.657
**Use of diagnosing techniques**			
	Preoperative ultrasound of the axilla	0.037	0.058
	Preoperative MRI	0.930	0.641
	Stereotactic guidance	0.019	0.299
	Vacuum-assisted device	0.274	0.231
**Decision to use SLNB is influenced by**			
	National guidelines	0.104	0.087
	Regional agreements	0.558	0.341
	Hospital agreements	0.955	0.096
	Multidisciplinary consultation	0.943	0.392
	Wish of the patient	0.730	0.936
	Own perception	0.909	0.457

For the influence of the national guideline, the details of the association are presented in the figures below.

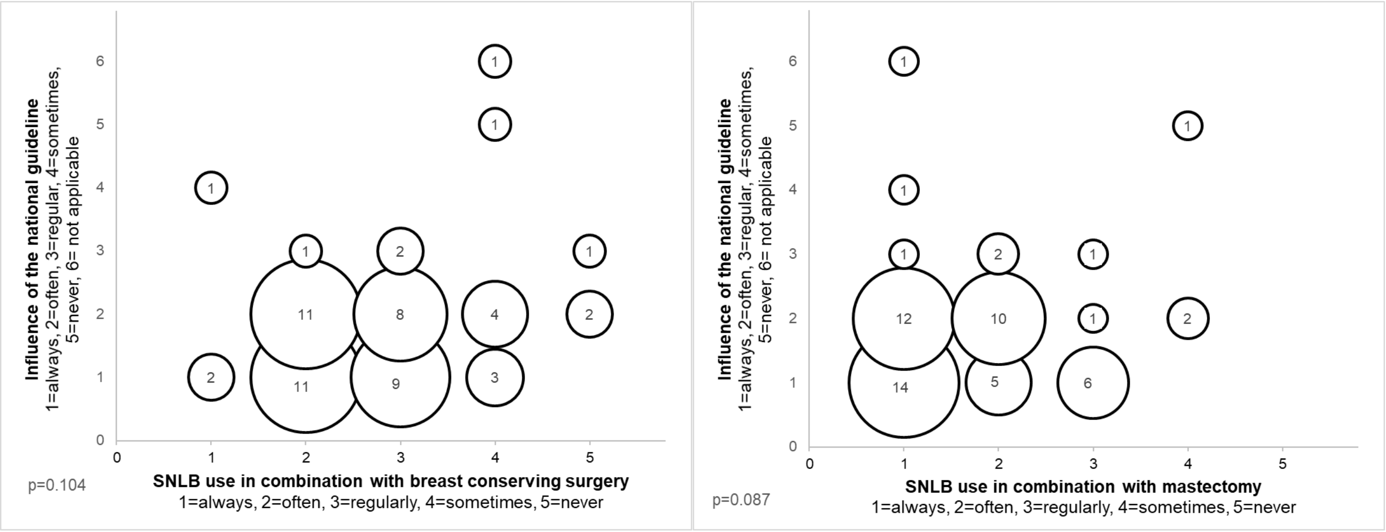

Supplement: S4 File — (PDF) [file pone.0269551.s004.pdf]
