# Supplementary material for: Surgeons’ preferences for using sentinel lymph node biopsy in patients with ductal carcinoma in situ
Source: PLoS One. 2022 Jun 6;17(6):e0269551. doi: 10.1371/journal.pone.0269551 (PMC9170095; doi:10.1371/journal.pone.0269551)
Supplement: S5 File — (PDF) [file pone.0269551.s005.pdf]

S5 Supplement:

guidelines and hospital patient information in the Netherlands

Guideline and online patient information assessed 2022, March

Dutch national guideline:

Dutch	English
Schildwachtklier procedure	*Sentinel lymph node biopsy*
Een SWK-procedure dient overwogen te worden bij*:*<br>• patiënten met preoperatieve diagnose DCIS voor wie een mastectomie is geïndiceerd in verband met de grootte.<br>• patiënten met een klein DCIS die in aanmerking komen voor MST, waarbij risicofactoren aanwezig zijn voor een invasieve component:<br>♦ jonger dan 55 jaar<br>♦ solide component op de mammografie<br>♦ toch suspectie op grond van de histologische biopten<br>♦ matig of slecht gedifferentieerd DCIS in biopten*.*<br>De SWK-procedure dient voorafgaand aan de resectie van het DCIS in dezelfde zitting plaats te vinden. Zeker bij een mastectomie is het in tweede instantie uitvoeren van een SWK-procedure minder betrouwbaar en daarom onwenselijk.	A SLNB should be considered for:<br>• patients with preoperative diagnosis DCIS for whom a mastectomy is indicated due to size of the DCIS.<br>• patients with a small DCIS who are eligible for breast conserving surgery, where risk factors are present for an invasive component:<br>♦ younger than 55 years<br>♦ solid component on the mammography<br>♦ suspicion on the basis of biopsy<br>♦ moderate or poorly differentiated DCIS<br>The SLNB must take place in the same session, prior to the resection of the DCIS. Especially in case of a mastectomy, performing the SLNB afterwards is less reliable and therefore undesirable.

Reference: https://www.lrcb.nl/resources/uploads/2017/02/Richtlijn-Mammacarcinoom.pdf

Update of the national guideline (valid 2022, March):

Dutch	English
Schildwachtklier procedure	*Sentinel lymph node biopsy*
Bij puur DCIS in de preoperatieve biopten kan een SWK-biopsie worden overwogen indien er risicofactoren op invasie aanwezig zijn. Een SWK-biopsie achteraf wordt niet aanbevolen.	In the case biopsy-proven DCIS, a SLNB may be considered if risk factors for invasion are present. A SLNB afterwards is not recommended.

Reference: https://richtlijnendatabase.nl/richtlijn/borstkanker/dcis/swk_biopsie.html

Examples of information hospitals have given online to patients on the treatment of DCIS and use of the sentinel lymph node biopsy for patients with biopsy-proven DCIS

Hospital: **Bravis Oncologie Centrum**

City: Roosendaal

Patient information:

Behandeling (Dutch)	Treatment (English)
Behandeling<br>De behandeling van een DCIS bestaat uit een operatie. Hierbij wordt het gebied met DCIS verwijderd met een marge omliggend weefsel. Afhankelijk van de uitgebreidheid van de DCIS en de grootte van de borst zal een advies voor een borstsparende operatie of het wegnemen van de borst worden gegeven.	*Treatment*<br>The treatment of DCIS consists of surgery, an excision of the area with DCIS with a margin of the surrounding tissue. Depending on the size of the DCIS and the size of the breast, an advice for breast conserving surgery or mastectomy will be given.
Verwijdering schildwachtklier<br>Omdat vooraf niet altijd duidelijk is of sprake is van een DCIS of DCIS met invasieve borstkanker, wordt ook de schildwachtklier procedure uitgevoerd. Dit vindt plaats als de kans op een invasieve vorm groot is:<br>• Bij een jonge vrouw<br>• Bij een groot gebied van DCIS (>5 cm)<br>• Bij een voelbare vorm van DCIS<br>• Bij DCIS graad 2 en DCIS graad 3	*Sentinel lymph node biopsy*<br>Because it is not always clear in advance whether there is a DCIS or DCIS with invasive breast cancer, the sentinel lymph node procedure is also performed. This is done when there is a high risk of invasive breast cancer:<br>• For young woman<br>• For a large area of DCIS (>5 cm)<br>• For palpable DCIS<br>• For DCIS grade 2 and for DCIS grade 3

Reference: https://www.bravisoncologiecentrum.nl/behandeling-van-dcis

Hospital: **Jeroen Bosch ziekenhuis**

City: 's-Hertogenbosch

Patient information:

Behandeling	*Treatment*
De behandeling bestaat uit een operatie, waarbij het gebied van de DCIS wordt verwijderd. Of u een borstsparende operatie of borst verwijderende operatie krijgt is afhankelijk van:<br>• hoe groot het DCIS gebied is<br>• de grootte van de borst<br>• en uw eigen voorkeur	The treatment consists of an operation, an excision of the area of the DCIS. Whether you will have breast conserving surgery or mastectomy is depending on:<br>• what size the DCIS area is<br>• the size of the breast<br>• and your own preference
Verwijdering schildwachtklier	*Sentinel lymph node biopsy*
Voor de operatie is niet altijd duidelijk of er alleen sprake is van DCIS, of van DCIS met borstkanker. Daarom wordt soms vóór de operatie van de borst de schildwachtklierprocedure gedaan.<br>Dit gebeurt als bij u een van de volgende punten van toepassing is:<br>• het gebied met DCIS groter is dan 5 centimeter<br>• de DCIS voelbaar is<br>• het gaat om DCIS van graad 3	Before the operation, it is not always clear whether there is only DCIS, or DCIS with breast cancer. Therefore, sometimes before the operation of the breast, the sentinel lymph node procedure is done.<br>This is done if you have one of the following:<br>• the area with DCIS is larger than 5 cm<br>• the DCIS is palpable<br>• it concerns DCIS grade 3

Reference: https://www.jeroenboschziekenhuis.nl/aandoeningen/voorstadium-van-borstkanker-dcis#group_modal_treatment

Hospital: **Zuiderland ziekenhuis**

City: Sittard

Patient information:

Behandeling<br>DCIS kan in de regel operatief op twee manieren worden behandeld, namelijk door middel van een borstsparende operatie of een borstamputatie. Deze beide behandelingen hebben een gelijke kans op genezing. Als er verschillende behandelopties zijn met gelijke kansen op genezing, dan is de keuze welke behandeling wordt uitgevoerd uiteindelijk aan u.	*Treatment*<br>There are two ways to treat DCIS surgically, by breast conserving surgery or a mastectomy. Both of these treatments have an equal chance of cure. If there are different treatment options with equal chances of cure, then the choice of treatment is ultimately up to you.
Verwijdering schildwachtklier<br>Soms moet de ‘schildwachtklierprocedure’ uitgevoerd worden. Deze vindt plaats als er een groot gebied van kalkspatjes is waarbij de kans aanwezig is dat er toch een invasieve component (borstkanker) bestaat.	*Sentinel lymph node biopsy*<br>Sometimes the 'sentinel lymph node procedure' has to be performed. This takes place when there is a large area of calcifications, since there is a risk of invasive breast cancer.

Reference: https://www.zuyderland.nl/online-folders/?guide=operatie-dcis-ductaal-carcinoma-in-situ
